# Central Superficial Quadriceps Tendon Harvest via Mini-Incision for Anterior Cruciate Ligament Reconstruction

**DOI:** 10.1016/j.eats.2025.103974

**Published:** 2025-11-01

**Authors:** Emmanuelle Yap, Bancha Chernchujit, Nishand Guruseelan

**Affiliations:** Department of Orthopedics, Faculty of Medicine, Thammasat University, Klong Luang, Thailand

## Abstract

Successful postoperative recovery among anterior cruciate ligament reconstruction patients constitutes both surgical reconstruction and donor-site morbidity. This article proposes a reproducible method using a mini-incision, thus decreasing donor-site morbidity and postoperative pain and among patients who undergo anterior cruciate ligament reconstruction. This technique does not rely on any integrated quadriceps tendon harvest guide systems while ensuring adequate graft size and length and preserving good quadriceps strength postoperatively.

Anterior cruciate ligament (ACL) reconstruction is one of the most commonly performed procedures in sports medicine. The success of this operation largely depends on graft selection, which impacts not only biomechanical stability but also donor-site morbidity, rehabilitation, and long-term outcomes. Among available autograft options—bone–patellar tendon–bone (BPTB), hamstring tendon (HT), and quadriceps tendon (QT)—the QT has gained increasing popularity as a reliable and versatile graft, particularly in the setting of primary and revision ACL reconstruction.^1^

The QT autograft offers several advantages. It has a larger cross-sectional area than patellar tendon and HT, and it shows favorable biomechanical characteristics, including high ultimate load to failure and stiffness.^2^ When compared with BPTB graft, QT has been associated with reduced anterior knee pain and kneeling discomfort.^3^ In contrast to HT graft, it preserves hamstring strength and may provide a more robust graft diameter, which is particularly advantageous in young athletes or patients with smaller HTs.^4^

Recent comparative studies have shown that clinical outcomes of QT autograft are equivalent or superior to those of BPTB and HT grafts in terms of knee stability, patient-reported outcome scores, and return-to-sport rates.^5^ Additionally, the risk of graft rupture appears to be comparable or lower, further supporting its use in high-demand populations.^6^

Our technique provides a graft of sufficient length and width while preserving the deep tendon fibers, which may reduce postoperative quadriceps weakness and extensor-mechanism problems. It avoids a bone block and specialized guide systems and is reproducible, efficient, and safe. Compared with standard quadriceps harvests (55-65 mm), we deliberately harvest a longer, partial-thickness central strip (240-300 mm) of the QT. The extra length allows double looping and tubularization to achieve a reliable 9- to 10-mm diameter for single-bundle ACL reconstruction. It also offers a comfortable working length for adjustable-loop fixation without graft-length concerns and keeps tunnel positioning flexible. The harvest is performed through a 2.5- to 3-cm mini-incision and can be reproduced even when specialized guide instruments are not available. This technical note outlines the indications, detailed surgical steps, and perioperative considerations of harvesting the central superficial QT using a mini-incision and discusses its role as an alternative to hamstring autograft in ACL reconstruction.

## Surgical Technique

### Patient Positioning

**Fig 1:**
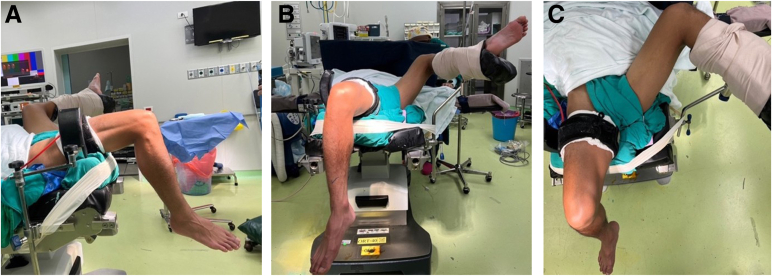
Patient positioning (right operative knee). The patient is placed in a supine, semi-lithotomy position, with the contralateral leg in a padded lithotomy holder (blue arrows); the operative knee is flexed to approximately 90° and supported over a bolster (red arrows). A high-thigh tourniquet (green arrows) is applied before preparation and draping, with exsanguination via Esmarch. (A) Lateral. (B) Anteroposterior. (C) Superior.

The patient is placed in a supine, semi-lithotomy position, with the contralateral leg abducted at the hip and placed into a well-padded lithotomy-style leg holder, ensuring it remains secure and out of the surgical field. The affected leg is flexed to 90° at the knee, and the posterior thigh is supported, allowing the leg to hang freely off the side of the table. This setup provides excellent exposure of the anterior thigh and distal quadriceps region while maintaining a relaxed quadriceps mechanism during graft harvest (Fig 1).

A high-thigh pneumatic tourniquet is applied on the operative limb and well padded to minimize the risk of tourniquet-associated neurapraxia. Once the patient is prepared and draped in standard sterile fashion, exsanguination is performed using an Esmarch bandage, followed by inflation of the tourniquet to the appropriate pressure (250 mm Hg) prior to skin incision. This positioning allows optimal access to the anterior thigh for QT harvest while providing sufficient clearance for arthroscopic instrumentation for ACL reconstruction.

### Skin Incision and Exposure

**Fig 2:**
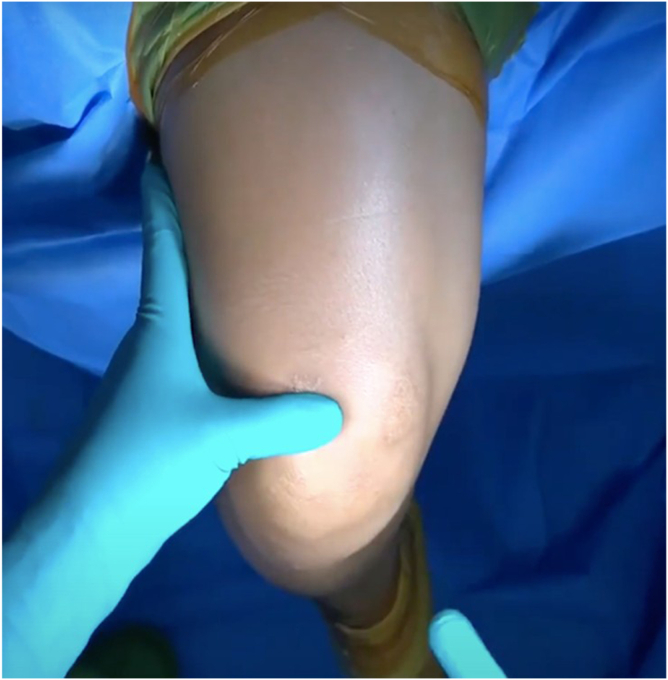
Skin landmarks and mini-incision. A 2.5- to 3-cm midline vertical incision (blue arrow, red dashes) is placed approximately 2 cm proximal to the superior pole of the patella (green arrow) over the central quadriceps tendon.

The procedure begins by identifying the superior pole of the patella and the midline of the distal anterior thigh. A vertical midline skin incision, approximately 2.5 to 3 cm in length, is made 2 cm (roughly 1 thumb breadth) proximal to the superior pole of the patella (Fig 2).

**Fig 3:**
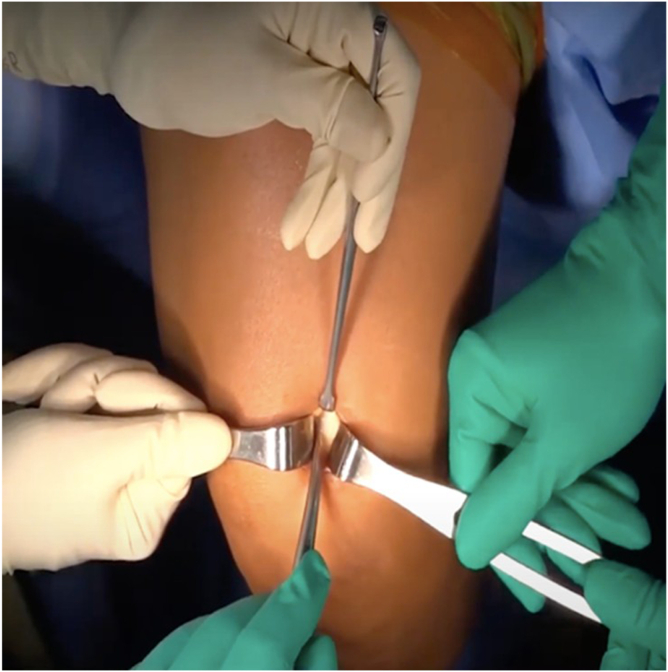
Blunt dissection to quadriceps tendon. Subcutaneous fat is gently cleared to expose the glistening quadriceps tendon fibers (blue arrow).

The incision is carried down through the dermis until the adipose layer is visualized. The fat is carefully separated by retractors and partially removed using blunt dissection. Once the adipose tissue is cleared from the surgical view, the white, glistening fibers of the QT become clearly visible (Fig 3).

The surgeon identifies the medial and lateral borders of the central QT, which define the boundaries of the superficial harvest. These borders serve as critical landmarks because they define the boundaries of the superficial QT. Adequate exposure of the tendon is essential to allow accurate marking of the harvest zone and to prevent unintended violation of surrounding structures.

### Tendon Identification and Graft Marking

**Fig 4:**
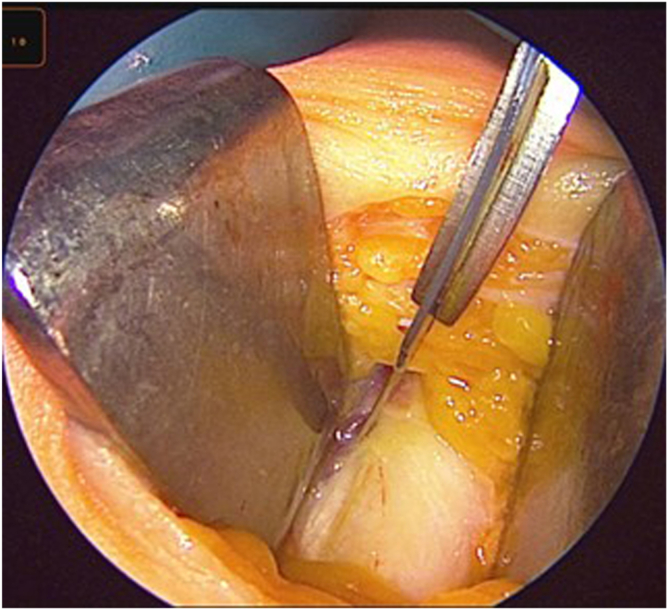
Central strip marking. Two parallel longitudinal cuts define a 1-cm-wide central strip (red double-headed arrow) along the rectus femoris–quadriceps tendon: lateral cut (blue line) and medial cut (green line). Depth is controlled to 6 to 8 mm.

With the QT fully visualized, attention is turned to marking the harvest zone. A No. 15 blade is used to make a longitudinal incision at the medial central edge of the rectus femoris. From this point, a second parallel incision is made approximately 1 cm lateral to define a 1-cm-wide central strip of the tendon (Fig 4). These incisions are made carefully and symmetrically to ensure proper graft geometry. They are made to a depth of approximately 6 to 8 mm, corresponding to the superficial layer of the QT.

### Partial-Thickness Harvest With Confirmation of Superficial Plane

The harvest is confined to the superficial 6 to 8 mm of the central QT. The correct depth is indicated by a smooth, glistening deep tendon layer remaining in situ with its longitudinal fibers visible and a thin sub-tendinous fat stripe separating the graft from the deep layer; gentle passage of a blunt dissector beneath the stripe should not cause the deep layer to move. The scissors or stripper tips must be kept parallel to the tendon and in constant contact with the undersurface of the strip during proximal release; the deep layer should remain flat and tensioned, with no muscle or synovium exposed. Loss of the fat stripe, brisk bleeding, or lifting of the deep layer with the graft suggests overly deep dissection; the surgeon should pause, re-establish the plane under direct vision, and continue superficially.

### Sub-tendinous Dissection and Distal Release

**Fig 5:**
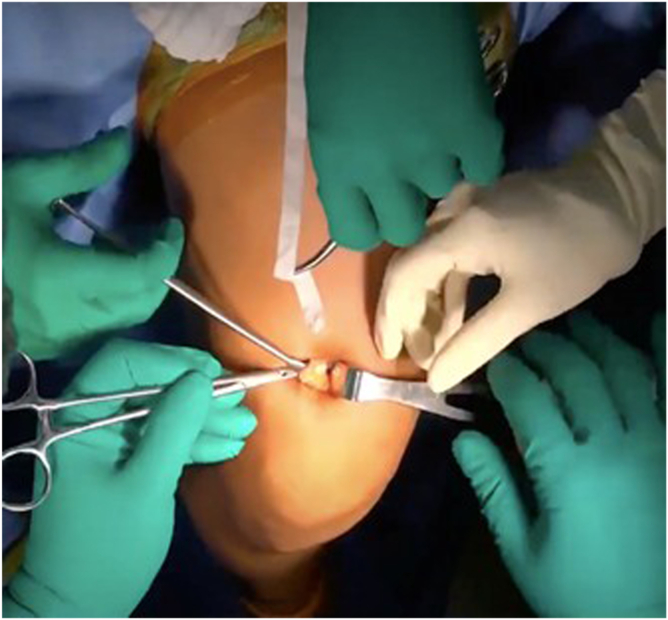
Sub-tendinous plane development. A right-angle forceps (blue arrow) passes beneath the superficial strip to establish the sub-tendinous plane, preserving the deep layer. The green arrow indicates surgical tape.

A right-angle forceps is gently introduced beneath the superficial tendon strip between the 2 incisions (Fig 5). This step must be performed delicately to avoid penetration of the deep tendon or damage to the rectus femoris muscle fibers.

**Fig 6:**
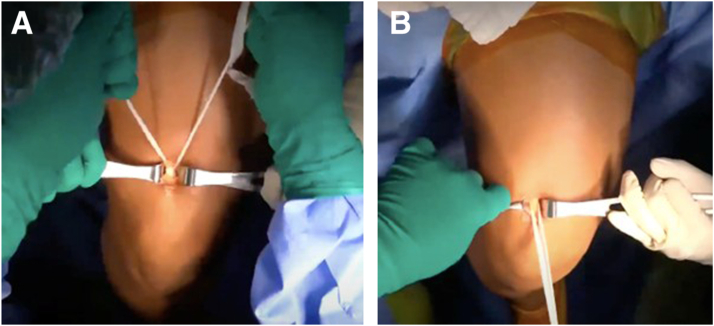
(A, B) Circumferential mobilization with tape. A sterile surgical tape (green arrows) is passed and gently moved in a sawing motion to release adhesions and complete mobilization.

Once the sub-tendinous plane is adequately developed, a surgical tape is passed under the tendon. The tape is gently moved in a sawing motion to further release any adhesions and facilitate circumferential mobilization of the graft (Fig 6). This maneuver enhances the freedom of the graft and reduces resistance during extraction.

**Fig 7:**
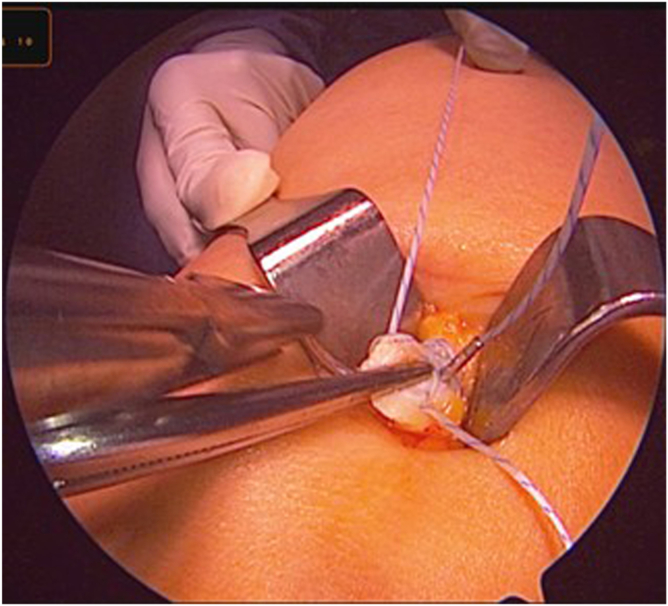
Distal control with whipstitch. After distal transection, a high-strength braided whipstitch (blue arrow) is applied to the graft end (red arrow) for traction.

With the tendon adequately released along its undersurface, the distal end is sharply transected at the distal window of the skin incision using a scalpel. Braided suture is then placed in a whipstitch configuration at the distal portion of the tendon (Fig 7).

### Proximal Mobilization and Graft Extraction

**Fig 8:**
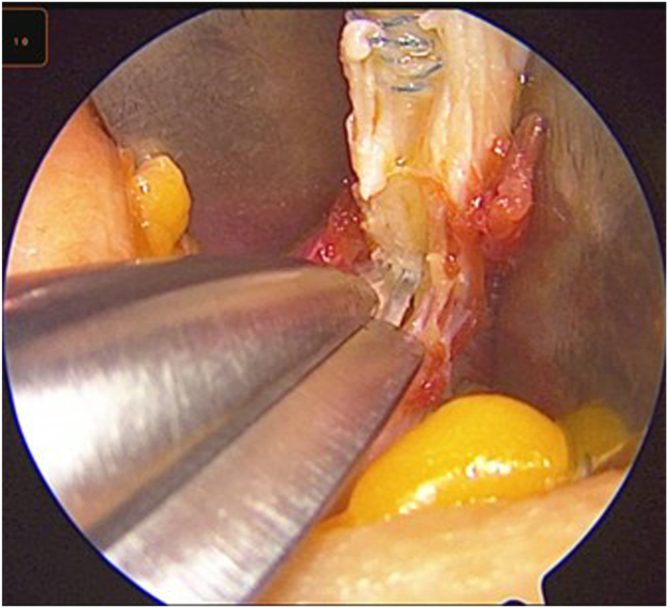
Arthroscopic-guided proximal dissection. Under arthroscopic visualization, Metzenbaum scissors (red arrow) extend the plane proximally while maintaining the superficial lamina (blue arrow).

After distal release and without a bone block, attention is directed to proximal mobilization. With gentle traction applied to the whipstitched distal end, Metzenbaum scissors are inserted into the sub-tendinous space to further dissect the proximal portion of the graft. With an assistant holding the arthroscope to guide visualization, deep dissection is continued, ensuring the correct plane and an approximate 1-cm width (Fig 8).

**Fig 9:**
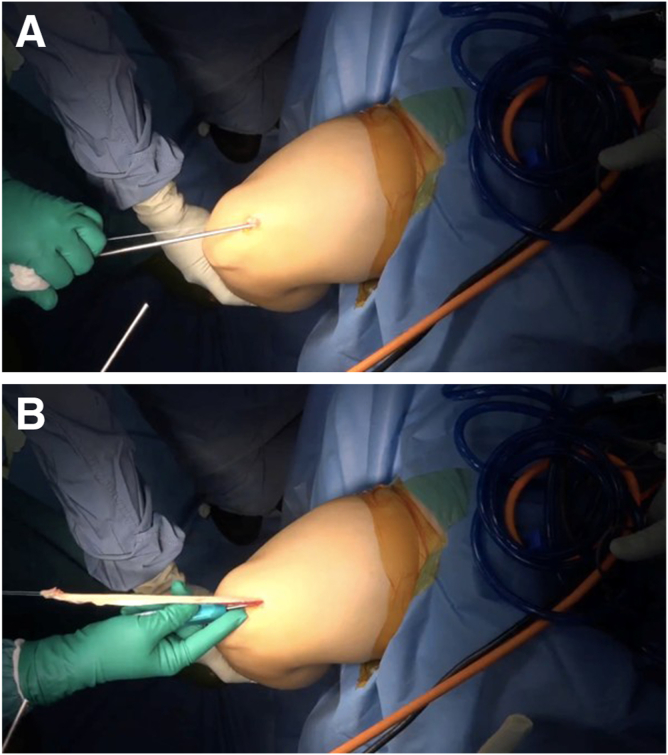
(A, B) Closed tendon stripper advancement. A 7-mm closed tendon stripper (blue arrows) is advanced in line with the tendon toward the anterior superior iliac spine until proximal release. The correct trajectory is shown (red arrows).

**Fig 10:**
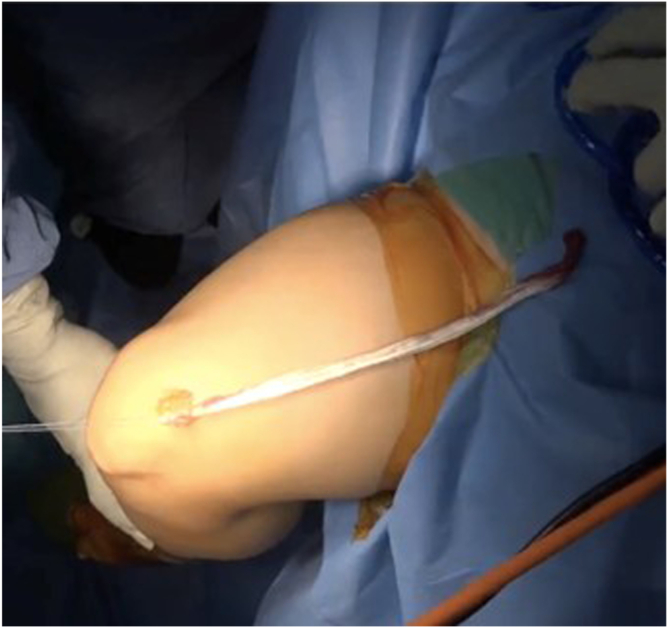
Harvested graft. The extracted central superficial quadriceps tendon graft is displayed on the thigh and measured; the typical length is 240 to 300 mm before preparation. The graft’s proximal and distal ends are labeled.

Once the graft is mobile, a 7-mm closed tendon stripper is introduced over the sutured end of the tendon. The tendon stripper is advanced proximally in line with the axis of the tendon, aiming toward the anterior superior iliac spine (ASIS) (Fig 9). The tendon stripper is advanced in a slow, controlled fashion until the graft separates proximally and is fully delivered through the incision; it is then placed on the thigh to estimate the length of the tendon from the distal incision (Fig 10). This technique is described in detail in Video 1.

### Graft Preparation

The harvested tendon is delivered through the incision and transferred to the back table for preparation. Residual soft tissue and muscle fibers are removed, and the graft is sized using a cylindrical sizing block. The graft diameter typically ranges from 9 to 10 mm, with an average length of 24 to 30 cm, depending on patient anatomy and harvest precision. This size is generally sufficient to accommodate most of our single-bundle ACL reconstruction techniques, providing both adequate length for fixation and robust graft volume for intra-articular reconstruction.

**Fig 11:**
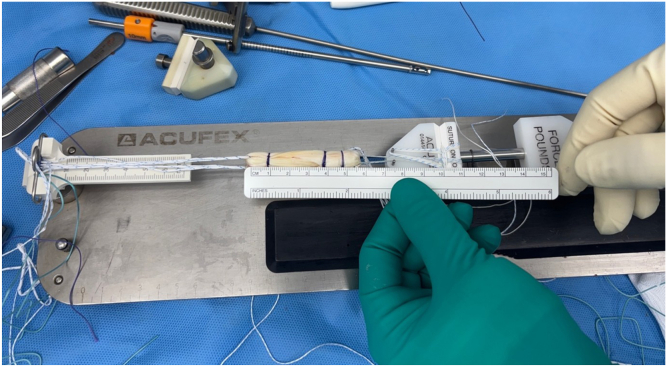
Graft preparation. On the preparation station, the graft is double looped and tubularized, with whipstitches at both ends and sizing to 9 to 10 mm. The graft is measured using a ruler (red arrow) to confirm the appropriate length.

On the preparation station, the graft is double looped and tubularized, and both ends are reinforced using whipstitch sutures with No. 2 high-strength braided suture material (Fig 11). These sutures allow for secure handling of the graft during passage and tensioning and serve as fixation points within the femoral and tibial tunnels.

### Closure

After graft harvest and preparation are complete, attention is turned back to the donor site. Hemostasis is achieved using diathermy electrocautery if necessary. The QT defect is typically left unrepaired to avoid excessive tension on the surrounding fibers.

The subcutaneous tissue is reapproximated with absorbable sutures. Skin closure is performed using interrupted nylon sutures. Sterile dressings are applied, and a compressive bandage is wrapped around the knee to reduce postoperative swelling and hematoma formation.

### Postoperative Rehabilitation

**Fig 12:**
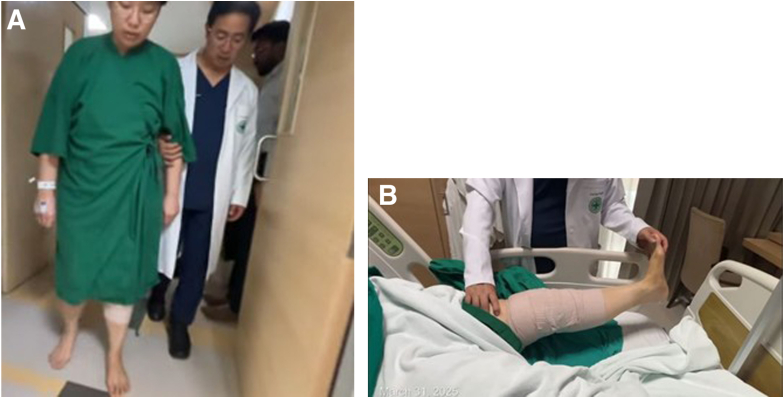
Early postoperative function. (A) Full weight-bearing ambulation with stable gait on postoperative day 1. (B) Early full range of motion is demonstrated without a hinged brace.

Patients are allowed to begin full range of motion and full weight-bearing without assistance immediately postoperatively (Fig 12A). A hinged knee brace is not routinely used because bracing may unnecessarily delay early mobilization and inhibit quadriceps muscle activation (Fig 12B). Emphasis is placed on early quadriceps strengthening, gait normalization, and progressive functional recovery. Most patients resume light activities by 3 months and return to pivoting or contact sports around 8 to 9 months, depending on clinical progress and functional milestones. A summary of key technical tips and commonly encountered pitfalls is provided in Table 1 to aid reproducibility and help avoid complications during the procedure.

**Table 1:** Tips and Pearls Versus Common Pitfalls

Tips and Pearls	Pitfalls
Make the incision 1-2 cm proximal to the superior pole of the patella.	An incision placed too low or too lateral limits exposure and affects the graft trajectory.
Limit the harvest to the superficial 6-8 mm of tendon.	Harvesting too deep may injure the rectus femoris or weaken the extensor mechanism.
Follow the tendon on the same plane of dissection when performing deep dissection using the sub-tendinous fat as a guide.	Forceful dissection may tear deep fibers or result in incomplete mobilization.
Use an arthroscope to guide the plane of deep dissection.	Poor plane identification can lead to an eccentric or asymmetrical graft harvest.
Advance the tendon stripper slowly, following the tendon axis toward the ASIS.	Aggressive or off-angle advancement may cause premature graft rupture.

ASIS, anterior superior iliac spine.

## Discussion

Our technique differs from standard QT harvests, which typically obtain a short segment (about 55-65 mm) and may include a bone block or a thicker, full-thickness strip taken with dedicated guides. In contrast, we harvest a long (240-300 mm), partial-thickness (6-8 mm) central strip through a mini-incision, preserving the deep tendon layer. Plane control is maintained under direct visualization and with a closed tendon stripper directed toward the ASIS. The added length allows reliable double looping and tubularization to achieve a 9- to 10-mm graft while maintaining the integrity of the extensor mechanism.

The rationale for a longer graft is practical. A strip of at least 240 mm provides a comfortable working length for preparation, tensioning, and passage; ensures an adequate intra-articular segment with secure purchase in both tunnels; and accommodates adjustable-loop fixation without graft-length concerns. In smaller patients with limited hamstring size, the longer QT harvest can eliminate the need for supplemental autograft and still permits augmentation if required.

The described approach offers several advantages (Table 2). The mini-incision reduces soft-tissue disruption, and the partial-thickness harvest preserves the deep layer, which may reduce postoperative quadriceps weakness. Avoiding a bone block may lessen anterior-knee and kneeling discomfort. The method is reproducible without guide systems, uses standard arthroscopy instruments plus a closed stripper, and consistently yields a robust graft that measures 9 to 10 mm in diameter. In our practice, early rehabilitation with immediate range of motion and full weight-bearing is feasible.

**Table 2:** Advantages and Disadvantages of Central Superficial QT Harvest (Mini-Incision)

Advantages
Mini-incision; low soft-tissue disruption
Partial thickness; deep layer preserved
No bone block, resulting in less anterior-knee pain or kneeling pain
Long graft (240-300 mm) enables 9- to 10-mm construct
Reproducible without guide systems
Early ROM and full weight-bearing feasible
Disadvantages/risks
Potential deep harvest, resulting in extensor weakness
Graft rupture if tendon stripper is off-axis
Donor-site hematoma; anterior thigh dysesthesia
Limited tendon caliber in small patients
Learning curve to maintain plane
Limited comparative outcomes available

QT, quadriceps tendon; ROM, range of motion.

There are, however, risks and limitations. Deeper dissection can weaken the extensor mechanism and should be mitigated by respecting the 6- to 8-mm depth and confirming the deep layer visually. Off-axis advancement of the stripper may jeopardize the graft; slow, in-line progression toward the ASIS is advised. Donor-site hematoma or dysesthesia can occur, and tendon caliber may be limited in very small patients. There is also a learning curve to maintaining the sub-tendinous plane. Given that this is a technical note without comparative outcomes, future studies should quantify quadriceps strength recovery, anterior-knee symptoms, graft failure rates, and return-to-sport timelines.

In settings in which hamstrings are small or BPTB morbidity is a concern, a long, partial-thickness QT graft obtained through a mini-incision provides a versatile, extensor-sparing autograft option that is practical in resource-variable environments. Overall, this technique is safe, efficient, and reproducible, delivering a long, durable graft while minimizing donor-site morbidity—thereby meeting contemporary needs in ACL reconstruction.

## Disclosures

All authors (E.Y., B.C., N.G.) declare that they have no known competing financial interests or personal relationships that could have appeared to influence the work reported in this paper.

## Supplementary Data

Video 1 Central superficial quadriceps tendon harvest via mini-incision. Step-by-step harvesting technique is presented, in addition to identification of important landmarks, development of the plane of dissection, and arthroscopic-guided dissection to minimize secondary skin incisions.
